# SLAB51 Multi-Strain Probiotic Formula Increases Oxygenation in Oxygen-Treated Preterm Infants

**DOI:** 10.3390/nu15173685

**Published:** 2023-08-23

**Authors:** Maria Elisabetta Baldassarre, Massimiliano Marazzato, Marta Pensa, Maria Teresa Loverro, Michele Quercia, Francesca Lombardi, Federico Schettini, Nicola Laforgia

**Affiliations:** 1 Section of Neonatology and Neonatal Intensive Care Unit, Department of Interdisciplinary Medicine, “Aldo Moro” University of Bari, 70121 Bari, Italy; mariaelisabetta.baldassarre@uniba.it (M.E.B.);; 2 Department of Public Health and Infectious Diseases, Sapienza University of Rome, 00185 Roma, Italy; 3 Department of Life, Health & Environmental Sciences, University of L’Aquila, 67100 L’Aquila, Italy; francesca.lombardi@univaq.it; 4 Neonatology and Neonatal Intensive Care, SS. Annunziata Hospital, 80058 Taranto, Italy

**Keywords:** probiotics, SLAB51, oxygen, hypoxia, hyperoxia, preterm infants

## Abstract

Preterm infants are at risk of hypoxia and hyperoxia because of the immaturity of their respiratory and antioxidant systems, linked to increased morbidity and mortality. This study aimed to evaluate the efficacy of a single administration of the SLAB51 probiotic formula in improving oxygenation in respiratory distress syndrome (RDS)-affected premature babies, thus reducing their need for oxygen administration. Additionally, the capability of SLAB51 in activating the factor-erythroid 2-related factor (Nrf2) responsible for antioxidant responses was evaluated in vitro. In two groups of oxygen-treated preterm infants with similar SaO_2_ values, SLAB51 or a placebo was given. After two hours, the SLAB51-treated group showed a significant increase in SaO_2_ levels and the SaO_2_/FiO_2_ ratio, while the control group showed no changes. Significantly increased Nrf2 activation was observed in intestinal epithelial cells (IECs) exposed to SLAB51 lysates. In preterm infants, we confirmed the previously observed SLAB51’s “oxygen-sparing effect”, permitting an improvement in SaO_2_ levels. We also provided evidence of SLAB51’s potential to enhance antioxidant responses, thus counteracting the detrimental effects of hyperoxia. Although further studies are needed to support our data, SLAB51 represents a promising approach to managing preterm infants requiring oxygen supplementation.

## 1. Introduction

The rate of premature births worldwide is continuously increasing, such that 5–18% of all deliveries are estimated to occur before the 37th week of gestation [1]. Perinatal care is necessary to avoid premature-birth-related complications and increase the survival rates of premature infants. Preterm babies often experience difficulties adapting to the extrauterine environment as they are characterized by an immature architecture of the lungs, thoracic cage immaturity, and surfactant deficiency within the lungs. To guarantee adequate oxygenation levels, a significant proportion of preterm infants usually need proactive interventions immediately in the delivery room and during the early period following birth while waiting for the respiratory system to become effective in guaranteeing optimal oxygenation.

The most significant complications of preterm birth are those associated with hypoxic and hyperoxic conditions. Hypoxia represents a physiological status in which peripheral tissues have unsatisfied oxygen demands due to an inadequate supply of this gas. Such a condition may arise from the inadequacy of the passive diffusion of oxygen from the alveolus to the pulmonary capillary, a low rate of oxygen transport from the lungs to peripheral tissues, and/or due to high oxygen consumption by tissues. Intermittent hypoxemia is mainly common in preterm infants during the postnatal period, probably due to immature respiratory control, resulting in apnea, respiratory pauses, and ineffective or obstructed inspiratory efforts in conjunction with several physiologic parameters, among which the most important is the pulmonary oxygen store. As a consequence of prolonged and/or intermittent hypoxia events, several morbidities may be encountered by preterm infants, including retinopathy of prematurity, sleep-disordered breathing, neurodevelopmental impairment, and also death [2,3].

Although oxygen supplementation in neonatal care represents the most commonly applied therapy to keep the saturation of preterm infants within the optimal range, such clinical practice is not free from harmful implications. Even if oxygen is mandatory for aerobic life, it plays a double-edged sword role in the perinatal period, responsible for benefits and toxic effects [4,5]. Oxygen toxicity is mainly due to the development of reactive oxygen species (ROS) and reactive nitrogen species (RNS), such as nitric oxide (NO), constituting potent oxidants in biological fluids and the leading causes of tissue damage through reactions with major biological macromolecules [6]. Since immature antioxidant capabilities are common in preterm infants, they show improved susceptibility to oxidative stress induced by high levels of free radicals, making them prone to oxidant injury, with short- and long-term effects at various body sites including, but not limited to, the lungs and retina [7,8]. In this context, it is important to underline that a high free radical production is induced by hypoxia and hyperoxia due to an excessive oxygen supply in organs and tissues. It is generally accepted that saturation targets adopted for children and adults are not considered suitable for preterm infants [9].

Despite several studies investigating the matter, there is no definitive evidence regarding the best values of SpO_2_ to use in the clinical management of premature newborns; thus, SpO_2_ targeting in preterm infants remains a controversial worldwide topic [10]. Ideally, the therapeutic goal in preterm infants is to achieve normoxia for the metabolic needs of the neonate while avoiding the consequences of both hypoxemia and hyperoxemia [11]. However, achieving optimal oxygen saturation in preterm infants by respiratory support is challenging as it depends on several factors, comprising the degree of pulmonary immaturity of the subject and the bedside caregiver’s ability to adjust FiO_2_.

The development of adjuvant therapeutic approaches able to exploit the physiological mechanisms which naturally determine the optimal oxygen saturation and protect from the harmful effects associated with hypoxia and hyperoxia would be desirable. In this context, targeting Hypoxia Inducible Factor 1 alpha (HIF1α) and the nuclear transcription factor-erythroid 2-related factor (Nrf2) could constitute a promising strategy [12,13]. HIF1α plays a pivotal role in regulating cell oxygen homeostasis and determining the body’s response to low oxygen concentrations. This factor is responsible for the activation of many genes that regulate diverse functions, including erythropoiesis, iron transport, angiogenesis, and glucose metabolism, which are prematurely inactivated in preterm infants due to the increased oxygen levels they are exposed to after birth. On the other side, Nrf2 is mainly responsible for activating genes involved in antioxidation, antioxidant biosynthesis, and metabolic shift, the stabilization of which could be used to combat hyperoxia-associated oxidative stress [14].

In recent studies, the administration of the multi-strain probiotic SLAB51 was associated with significantly higher blood levels of both the partial pressure of oxygen (pO_2_) and the arterial oxygen saturation (SaO_2_) in adult COVID-19 patients with pneumonia treated with non-invasive ventilation [15,16]. These results highlight the ability of this multi-strain probiotic formulation to influence the redistribution of oxygen throughout the body through a possible oxygen-sparing effect involving the stabilization of HIF1α at the intestine level, in agreement with results published by Lombardi and colleagues [17]. Furthermore, Slab51 has previously been proven to exert an antioxidant effect as well as to restore Nrf2 levels and its main target Haem oxygenase-1 gene (HO-1) in an animal model of Parkinson’s disease [18,19]. HO-1 is an inducible enzyme exhibiting antioxidant and anti-inflammatory properties [20].

The primary aim of our study was to assess whether a single administration of SLAB51 in preterm neonates could have the same effect observed in adults by improving oxygenation and reducing the need for supplemental oxygen. Additionally, the capability of SLAB51 in influencing Nrf2 stabilization in the intestine, thus potentially favoring an antioxidant effect, has been explored in vitro on the human intestinal epithelium (intestinal epithelial cells, IECs).

## 2. Materials and Methods

### 2.1. Pilot Sample

This was a single center, double-blind, placebo-controlled trial in which both the parents of premature neonates and the experimenters responsible for administering the treatment and evaluating oxygen saturation were kept blinded. Thirty preterm neonates with oxygen demand after respiratory distress syndrome (RDS) or (early) bronchopulmonary dysplasia treated with non-invasive ventilation (HFT: high-flow therapy) or invasive ventilation (assisted controlled ventilation, A/C, and synchronized intermittent mandatory ventilation, SIMV) were included in the study. Exclusion criteria were the presence of congenital malformations or surgical conditions. Clinical data, i.e., gestational age, auxological data at birth and at the enrollment, Apgar score, feeding type, and ventilation modes, were collected from electronic medical charts. Due to the unavailability of a bank of human donor milk in the center responsible for the study, each enrolled subject was fed with fresh breast milk taken from the mother in combination, when needed, with fed formula appropriate for the weight and gestational age or for previously manifested food intolerance problems (hydrolyzed milk). Preterm neonates were randomly assigned to the treated group (TG, n = 15) or the control group (CG, n = 15), alternating the allocation of subjects to the two groups according to the enrollment sequence. The TG received SLAB51 multi-strain probiotic formulation orally, at 1 billion total bacteria per kg of weight. SLAB51 contains *Streptococcus thermophilus* CNCM I-5570, *Bifidobacterium lactis* CNCM I-5571, *Bifidobacterium lactis* CNCM I-5572, *Lactobacillus acidophilus* CNCM I-5567, *Lactobacillus helveticus* CNCM I-5573, *Lactobacillus paracasei* CNCM I-5568, *Lactobacillus plantarum* CNCM I-5569, and *Lactobacillus brevis* CNCM I-5566. The CG received a placebo orally. The probiotic formulation and the placebo contained the same ingredients, except for the bacterial strains not present in the placebo. The two products were identical in appearance and taste. Probiotic administration was carried out by dissolving a single capsule containing 10 billion live bacteria in 10 mL of physiological solution and administering the treated subject with a volume in ml equal to weight in Kg registered immediately before the beginning of the treatment. The same procedure was used for the placebo. SaO_2_, FiO_2_, and the SaO_2_/FiO_2_ ratio were measured 2 h before administration (−2 h), immediately before product administration (0 h), and 2, 4, and 6 h after administration. SaO_2_/FiO_2_ is an effective and non-invasive tool to evaluate the severity of RDS [9]. Since the premature infants included in the study showed SaO_2_ levels free from relevant fluctuations in the days preceding the start of treatment, during the evaluation period, no changes in the ventilatory setting were allowed, with no increased risk to the health of the subjects. FiO_2_ was then reduced by 5% if SaO_2_ exceeded 97%.

### 2.2. In Vitro Study

#### 2.2.1. Preparation of Bacterial Lysate for Cell Treatments

The bacterial lysate was prepared as previously described [17]. Briefly, the SLAB51 formulation was suspended at a concentration of 133 × 10^9^ CFU in 10 mL of phosphate-buffered saline (PBS, Euro Clone, West York, UK), centrifuged at 8600× *g*, washed twice, and sonicated (30 min, alternating 10 s of sonication and 10 s of pause) using a Vibracell sonicator (Sonic and Materials, Danbury, CT, USA). Bacterial cell disruption was verified by measuring the absorbance of the sample at 590 nm with a spectrophotometer (Eppendorf Hamburg, Germany) before and after every sonication step. The samples were then centrifuged at 17,949× *g*, and the supernatants were filtered using a 0.22 µm pore filter (Corning Incorporated, Corning, NY, USA) to remove any whole remaining bacteria. The total protein content was determined by a DC Protein Assay (BioRad, Hercules, CA, USA) using bovine serum albumin (BSA, Sigma Aldrich, St. Louis, MO, USA) as the standard.

#### 2.2.2. Caco-2 IECs

The Caco-2 cell line, purchased from Sigma-Aldrich (St. Louis, MO, USA), was grown in tissue culture flasks at 37 °C, 5% CO_2_, and 90% relative humidity. The culture medium (Dulbecco’s modified Eagle’s medium, DMEM), supplemented with 10% fetal calf serum (FCS), 1% non-essential amino acids, 1 mM sodium pyruvate, 2 mM L-glutamine, 100 U/mL penicillin, and 100 μg/mL streptomycin (Euro Clone, West York, UK), was refreshed every other day. After reaching 80% confluence, cells were detached with trypsin solution from bovine pancreas (Euro Clone, West York, UK), seeded into sterile tissue culture 6-well plates (Becton, San Jose, CA, USA) at 60,000 cells/cm^2^, and the cell growth was monitored via microscopy. Fourteen days post-confluence, cells were incubated with or without the SLAB51 lysate at the indicated concentrations. No significant influence on the cell viability compared to the control cells was registered after the treatment with SLAB51, as evaluated by a Trypan blue assay.

#### 2.2.3. Western Blot

Cells were washed with cold PBS and removed from plates by scraping in RIPA Lysis Buffer (Merck KGaA, Darmstadt, Germany) containing 100 mM of a protease inhibitor cocktail (Sigma-Aldrich, St. Louis, MO, USA). After lysis, the samples were centrifuged at 17,949× *g* to eliminate cell debris. The supernatants were collected and assayed for protein content with a DC Protein Assay. Then, 25 µg of total proteins was resolved in 10% sodium dodecyl sulphate–polyacrylamide gel electrophoresis (SDS-PAGE) and electroblotted onto nitrocellulose membranes (Bio-Rad Laboratories, Hercules, CA, USA). Membranes were blocked with 5% non-fat dry milk for 1 h at room temperature and then incubated overnight at 4 °C with rabbit monoclonal antibody anti-Nrf2 (phospho S40) (Abcam, Cambridge, UK) 1:1000, or with mouse monoclonal antibody anti-β-actin 1:1000 (OriGene Technologies, Inc, Rockville, MD, USA). Horseradish peroxidase (HRP)-conjugated goat anti-rabbit IgG secondary antibody (Millipore EMD, Darmstadt, Germany) 1:2000 or HRP-conjugated goat anti-mouse IgG secondary antibody (Bio-Rad Laboratories) 1:2000 was used. Immunoreactive bands were visualized by enhanced chemiluminescence (ECL, Amersham Pharmacia Biotech, Erie, PA, USA) according to the manufacturer’s instructions. Band relative densities were determined using a chemiluminescence documentation system (Alliance; UVITEC, Cambridge, UK) and values were given as relative units.

### 2.3. Statistical Analysis

A descriptive data analysis was performed using tables and graphs corresponding to the qualitative or quantitative variables. The Shapiro–Wilk test was used to evaluate the normality of the data. A sample size calculation was not performed. The categorical variables were compared using the χ^2^ test with Yates’ continuity correction and reported as absolute frequencies and percentages. Unpaired and paired Student’s *t*-tests were performed to determine significant differences between the two groups and, for each group, between different time points, respectively. Where necessary, the computed *p* values were adjusted using the Benjamini–Hochberg procedures for accounting for multiple comparisons. For the in vitro study, a comparison of the mean values among the groups was performed by using a one-way ANOVA, followed by a Dunnett’s or Tukey’s post hoc test. The results were expressed as means ± SEM, as specified in figure legends. In each case, *p* ≤ 0.05 was considered statistically significant. Statistical analysis was performed using SPSS version 25.0 (IBM, Armonk, NY, USA).

## 3. Results

We did not find any significant differences between the two groups at birth and enrollment, as reported in Table 1.

**Figure 1:**
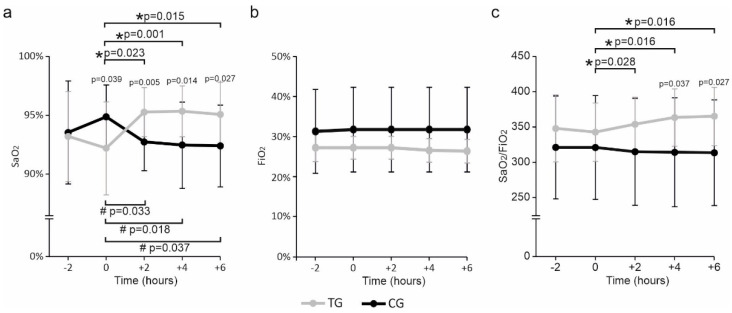
Color-coded line plots are relative to (**a**) SaO_2_, (**b**) FiO_2_, and (**c**) the SaO_2_/FiO_2_ ratio. Values are reported as means ± SD. *p* values reported above error bars relate to the statistical significance between the two groups of patients at each time point. In contrast, significant values between different time points are highlighted by horizontal bars designated by * and # for the treated and control groups, respectively. A *p*-value ≤ 0.05 was considered statistically significant in each case. TG: treated group; CG: control group.

Two hours before administration (−2 h), the TG and the CG had similar SaO_2_ values (Figure 1a). At 0 h, immediately before the administration, SaO_2_ decreased in the SLAB51 group but increased in the control group compared to −2 h, leading the two groups to show significantly different SaO_2_ values in favor of a higher level in the CG than in the TG. Two, four, and six hours after the administration, SaO_2_ was significantly increased with respect to the baseline in TG but reduced considerably in CG.

Significantly higher SaO_2_ values in the TG than the CG were already evident at 2 h and persisted up to 6 h. FiO_2_ levels did not differ between groups at any time assessed, although lower values were registered for TG than CG (Figure 1b). In agreement with what has previously been reported for oxygen saturation levels, significant increases in the ratio were observed for the SLAB51 group at 2, 4, and 6 h relative to the baseline. Although not statistically significant, there was a tendency for the SaO_2_/FiO_2_ ratio to decrease relative to the baseline in the control group (Figure 1c).

At 4 and 6 h, the ratio of SaO_2_/FiO_2_ was significantly greater in the SLAB51 group than in the control group. Any modifications in gastrointestinal tolerance during and after administration were reported in both groups.

**Figure 2:**
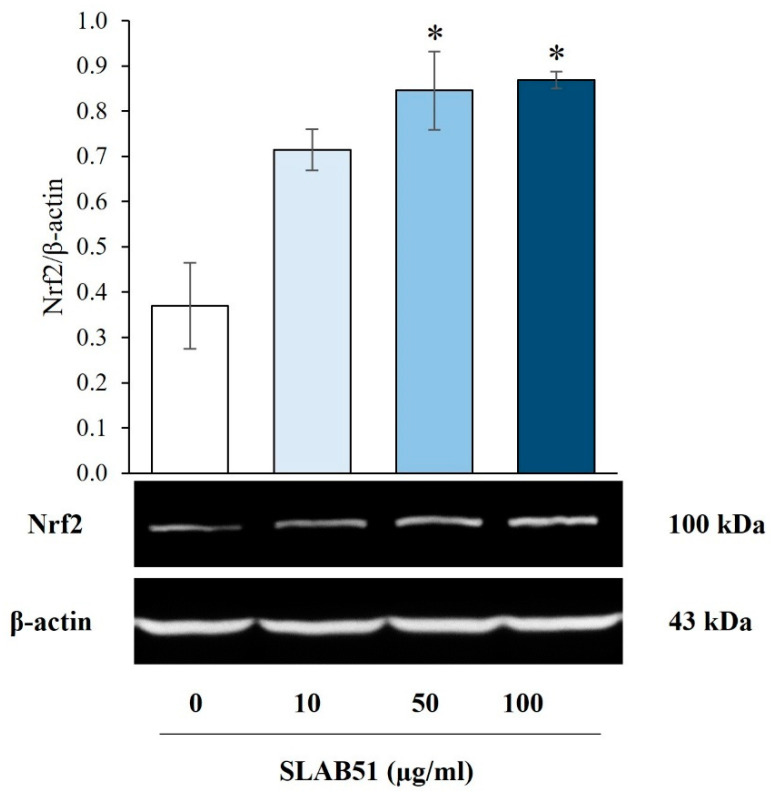
Effect of SLAB51 lysate on Nrf2 levels in IECs. Cells were incubated with increasing concentrations of SLAB51 bacterial lysate for 24 h; Nrf2 levels were then evaluated by Western blotting. Following the densitometric analysis, the obtained values were normalized to β-actin. Values are expressed as means ± SEM of two independent experiments. Representative immunoblots are also shown. A one-way analysis of variance (ANOVA) followed by Dunnet’s test was used for the comparative analysis of the data. * *p* < 0.05 vs. untreated cells.

The in vitro analysis assessing the effect of SLAB51 on cellular Nrf2 revealed that IECs exposed to bacterial lysates at concentrations of 50 and 100 µg/mL presented significantly higher Nrf2 levels than the untreated control. Moreover, although not statistically significant, an increased expression of this factor was also observed while exposing cells to the lower lysate concentration (10 µg/mL) (Figure 2).

## 4. Discussion

As a result of an immature lung architecture, underdeveloped thoracic cages, and insufficient surfactants, preterm infants frequently experience hypoxemic conditions from birth, requiring clinical interventions to achieve adequate oxygenation [11]. Although oxygen supplementation represents the most common approach to achieving optimal oxygen saturation in preterm newborns, this practice may have detrimental consequences, mainly related to the risk of hyperoxemia. This condition is associated with the production of reactive oxygen species that react with lipids, proteins, and nucleic acids and may cause tissue damage as a result of the inability of premature infants to respond to such insults due to the immaturity of their antioxidant response [6,8].

It is well known that blood flow in the intestines is subjected to deep fluctuations, increasing during digestion and absorption of nutrients and reducing during physical exercise. It is also noteworthy that the intestine has physiological mechanisms permitting this body area to tolerate hypoxia. In the short term, redistributing the oxygen from the intestines to other critical organs constitutes a strategy that may enhance the infant’s survival, like turning off the water in the garden to have more water pressure in the house. This “redirection” could be the solution to increase oxygen saturation levels when extensive alveolar damage is present and where a further increase in the amount of oxygen administered does not offer additional benefits and may even create serious side effects. Since this effect is physiologically driven, the additional amount of oxygen available in the bloodstream will be finely controlled, thus not exceeding the body’s natural demands and drastically reducing the risk of hyperoxia often associated with the external supply of oxygen.

Probiotics have extensively been used safely in preterm infants with controversial results [21]. These microorganisms reach the colon in a viable state, interact with the residing microbiota, and colonize that organ to exert their beneficial effect. Most studies have focused on developing healthy microbiota through specific metabolic activities in the colon. Probiotics can also release substances or induce direct responses from the host cells by influencing specific metabolic pathways and could also positively impact body sites other than the colon due to communication axes linking the colonic microbes with other organs and tissues [22,23,24]. Studies on adult COVID-19 patients with pneumonia have shown that administering multi-strain probiotics, i.e., SLAB51, could increase oxygenation [15,16]. Since the effect on oxygen saturation generally occurs as early as 2–3 h after ingestion, while any ingested food takes 6–8 h to reach and pass the ileocecal valve, it has been postulated that SLAB51 exerts its action in the intestine before colonizing the colon. In this context, probiotic strains contained in the SLAB51 formulation may establish direct crosstalk with various host cell types, including host intestinal cells, influencing the latter’s activity through their enzymatic capabilities [15,25].

The SLAB51 formulation contains bacteria expressing ADI activity. The formulation could promote vasoconstriction by limiting NO production at the mucosal level and, consequently, the amount of oxygenated blood reaching the intestine [15]. NO constitutes one of the primary regulators of vascular tone and tissue perfusion through gut mucosal vasodilation and one of the significant factors modulating the activity of prolyl-4-hydroxylases (PHDs), enzymes responsible for the hydroxylation of hypoxia-inducible factors (HIFs) at proline residues. Hydroxylation serves as an interaction scaffold for recognizing the Von Hippel–Lindau (VHL)-containing E3-ligase complex and the subsequent degradation of HIFs by the proteasome. PHDs present overlapping but peculiar tissue expression patterns, with a different affinity for inhibiting particular HIFα subunits. Within the PHD family, PHD2 is considered the most crucial oxygen sensor during normoxia and mild hypoxia, displaying a stronger preference for HIF1α as a substrate [26]. The activity of PHDs is regulated at various levels, including transcription and post-translation, and have a direct influence on their catalytic activity. By regulating mitochondrial oxygen consumption, NO affects the overall HIF response, influencing the activity of PHDs and subsequently inducing the degradation of alpha subunits [27]. Stabilizing HIFs, particularly HIF1-α, protect intestinal cells from the harmful effects of short-term hypoxia exposure [28].

HIFs are critical in regulating cellular oxygen homeostasis and determining the body’s response to low oxygen concentrations. Among HIFs, HIF-1α is constitutively produced at high levels in most cell types and mediates various hypoxia-associated responses, such as glucose uptake, anaerobic respiration, angiogenesis, oxidative stress response, and erythropoiesis [12,29,30]. When adequate oxygen levels are present, HIF-1α subunits are continuously hydroxylated by PHDs, while under hypoxic conditions, PHD activity is limited, permitting HIFs to accumulate and subsequently be transferred to the cell nucleus, where dimerizing with HIF1β subunits promotes the activation of genes responsible for adaptation to hypoxia [27].

The modulation of HIF and PHD activities is particularly interesting for treating hypoxemic preterm infants [30]. It has also been reported that pharmacological inhibition of PHDs and the subsequent stabilization of HIF1-α can prevent retinopathy in preterm infants [12]. A recent experimental study on a murine model demonstrated that SLAB51 administration is associated with reduced NO production, increased HIF1-α levels, and reduced PHD2 expression [24]. This effect has been further observed in a recent in vitro study evaluating the impact of SLAB51 on intestinal epithelial cells, showing a dose-dependent increase in HIF-1α protein intracellular amounts due to a significant reduction in PHD2 levels and activity [17]. Significantly higher glycolytic metabolism and L-lactate production were also registered, meaning that SLAB51 may influence the cell’s energy metabolism, directing it toward oxygen-independent metabolic pathways. A similar mechanism by SLAB51 in the intestine can reduce local oxygen consumption, allowing a redistribution of the available oxygen to other more critical organs. Our data have shown that a single administration of SLAB51 increases oxygenation in preterm infants needing oxygen support, confirming data found in adult COVID-19 pneumonia patients [15].

As a further result of our study, the SLAB51 formulation has been proven to act on intestinal cells by increasing activation of Nrf2, which is commonly involved in the transcriptional regulation of genes encoding antioxidant proteins under stress conditions, particularly in the protection against oxidative stress in the retinal pigment epithelium [31]. The capability of SAB51 in activating Nrf2 is supported by recent findings published by Lombardi et al. in 2023, showing that the same formulation positively modulates the activation of PI3Kinase/AKT signaling [17]. Such a pathway is responsible for many biological responses, including HIF-1α stabilization but also Nrf2 activation and the subsequent dampening of hyperoxia-induced detrimental effects such as lung injuries [32]. In this context, SLAB51 not only provides improvements in the management of oxygenation of premature newborns, but it can potentially constitute a valid approach for reducing the risks associated with the hyperoxia conditions often related to external oxygen supplementation.

The rapid onset of the “oxygen-sparing effect” of SLAB51 could result from a direct interaction between the bacterial strains in the formulation and the host cells in the proximal part of the intestine and not at more distal intestinal regions such as the colon. If our results of significant improvements in SaO_2_ and the ratio of SaO_2_/FiO_2_ are confirmed in a higher number of preterm infants, SLAB51 could represent a possible adjunctive therapy in preterm infants with RDS who need ventilation and oxygen supplementation. There are notable limitations to this study. First, the study included a small number of infants. An additional limitation of our study is the length of observation of only six hours post-administration, a limited time to evaluate if the improvement of oxygenation lasts longer or if subsequent doses of SLAB51 could maintain it. A further limitation of the study concerns the fact that the involvement of the NRF2 pathway constitutes only one theoretically possible explanation for the effect seen in vivo, while other effects may be more relevant. A randomized, double-blind, placebo-controlled trial with a proper sample size calculation is undoubtedly needed to confirm our findings. Furthermore, the expansion of the study to longer times and to the administration of successive doses of SLAB51 could provide stronger evidence on the dynamics of action of this probiotic therapy and on its clinical efficacy in improving oxygenation in premature newborns.

## Figures and Tables

**Table 1:** Clinical data relative to preterm infants enrolled in the study and stratified in agreement with administering the probiotic formula SLAB51.

Clinical Variable	TG (No. 15)	CG (No. 15)	*p* Value
Gestational age at birth (weeks) (mean ± SD)	29.7 ± 4.1	27.7 ± 3.6	0.16
Gestational age at enrollment (weeks) (mean ± SD)	33.3 ± 4.1	31.5 ± 5.1	0.43
Gestational age at enrollment <32 weeks (no. -%)	10–66.7%	14–93.3%	0.17
Weight at birth (kg) (mean ± SD)	1.6 ± 0.89	1.1 ± 0.72	0.11
Weight at enrollment (kg) (mean ± SD)	1.9 ± 0.82	1.5 ± 0.95	0.24
Apgar score			
1 min	6.6 ± 2.5	5.4 ± 1.7	0.14
5 min	8.0 ± 1.6	8.0 ± 0.84	1
Feeding (no. -%)			
Breastfeeding	0–0.0%	1–6.7%	1
Breastfeeding + preterm formula	11–73.3%	11–73.3%	0.68
Breastfeeding + formula type 1	1–6.7%	1–6.7%	0.46
Breastfeeding + hydrolyzed formula	3–20.0%	1–6.7%	0.59
Total parenteral nutrition	0–0.0%	1–6.7%	1
Ventilation mode (no. -%)			
A/C	0–0.0%	1–6.7%	1
HFT	15–100.0%	12–80.0%	0.22
SIMV	0–0.0%	2–13.3%	0.46

A/C: assisted controlled ventilation; HFT: high-flow therapy; SIMV: invasive ventilation and synchronized intermittent mandatory ventilation.

## Data Availability

Data regarding investigated patients and analytic methods will be made available on request.
